# Regulation of Early Lymphocyte Development *via* mRNA Decay Catalyzed by the CCR4-NOT Complex

**DOI:** 10.3389/fimmu.2021.715675

**Published:** 2021-07-19

**Authors:** Taishin Akiyama, Tadashi Yamamoto

**Affiliations:** ^1^ Laboratory for Immune Homeostasis, RIKEN Center for Integrative Medical Sciences, Yokohama, Japan; ^2^ Graduate School of Medical Life Science, Yokohama City University, Yokohama, Japan; ^3^ Cell Signal Unit, Okinawa Institute of Science and Technology Graduate University, Okinawa, Japan

**Keywords:** mRNA decay, CCR4-NOT complex, lymphocyte development, Apoptosis, VDJ recombination

## Abstract

Development of lymphocytes is precisely regulated by various mechanisms. In addition to transcriptional rates, post-transcriptional regulation of mRNA abundance contributes to differentiation of lymphocytes. mRNA decay is a post-transcriptional mechanism controlling mRNA abundance. The carbon catabolite repression 4 (CCR4)-negative on TATA-less (NOT) complex controls mRNA longevity by catalyzing mRNA deadenylation, which is the rate-limiting step in the mRNA decay pathway. mRNA decay, regulated by the CCR4-NOT complex, is required for differentiation of pro-B to pre-B cells and V(D)J recombination in pro-B cells. In this process, it is likely that the RNA-binding proteins, ZFP36 ring finger protein like 1 and 2, recruit the CCR4-NOT complex to specific target mRNAs, thereby inducing cell quiescence of pro-B cells. A recent study showed that the CCR4-NOT complex participates in positive selection of thymocytes. Mechanistically, the CCR4-NOT deadenylase complex inhibits abnormal apoptosis by reducing the expression level of mRNAs encoding pro-apoptotic proteins, which are otherwise up-regulated during positive selection. We discuss mechanisms regulating CCR4-NOT complex-dependent mRNA decay in lymphocyte development and selection.

## Introduction

Pleiotropic mechanisms control cytoplasmic mRNA abundance. Besides transcriptional regulation, post-transcriptional mechanisms are critical for controlling the level of cytoplasmic mRNA. mRNA decay is a post-transcriptional mechanism for reducing mRNA abundance. One major role of mRNA decay systems is to control homeostatic turnover and quality of mRNA. In addition, mRNA decay pathways actively regulate mRNA abundance, which is necessary to maintain and alter mRNA quantity in response to physiological signals. Many studies have suggested that active regulation of mRNA decay mechanisms must be critical for immune regulation and homeostasis (1–14). In this review, we focus on functions of the mRNA decay system regulated by the carbon catabolite repression 4 (CCR4)-negative on TATA-less (NOT) deadenylase complex in early lymphocyte development.

## mRNA Decay Mechanisms

Functionally, mRNA decay comprises two classes. The first is the mRNA decay system required for RNA surveillance to prevent generation of potentially toxic proteins (15). Nonsense-mediated mRNA decay (NMD) is an mRNA quality control pathway that degrades aberrant mRNAs with premature termination codons (16–18). In addition, No-go decay and No-stop decay pathways lead to mRNA decay in cases of ribosome stalling due to accidental blockades of translation and failure of termination, respectively (19).

The second class is the mRNA decay system that actively regulates amounts of mRNA encoding functional proteins. Exonuclease and endonuclease mRNA decay pathways mainly contribute to this “active” mRNA decay (14, 16, 20). In addition, some recent studies have proposed involvement of the NMD mechanism in regulation of mRNAs encoding full-length proteins during embryonic development and tissue-specific cell differentiation (18, 21).

## Initiation Of The mRNA Decay Pathway By Deadenylation

The exonuclease pathway of mRNA decay is initiated by removing polyA tails from mRNAs (16, 20). Following deadenylation of polyA tails, the 5’ cap structure of deadenylated mRNAs is removed by recruiting the decapping complex (Dcp1/Dcp2). Then 5’-3’ exoribonuclease 1 and 2 degrade decapped mRNAs from their 5′ ends.

Deadenylation of mRNA is the rate-limiting step for the exonuclease pathway. At present, three deadenylases have been reported: carbon catabolite repressor 4-negative on the TATA (CCR4-NOT) complex, polyA nuclease 2 (Pan2)-Pan3, and polyA-specific ribonuclease (PARN) (16, 20). A previous study suggested that cytoplasmic deadenylase activity of the CCR4-NOT complex predominated (22). In contrast, PAN2/3 trims relatively long tails of polyA (above 150 nt) and exerts minimal influence on the transcriptome.

In humans and mice, The CCR4-NOT complex is composed of eight protein subunits (23–25). CCR4-NOT transcription complex subunit 1 (CNOT1) serves as a scaffold to assemble the other subunits and recruits RNA-binding proteins (26–28). Two subunits, CNOT6/6L and CNOT7/8, have deadenylase activity (29–32). Other CNOT subunits (CNOT2, 3, 9,10, and 11) lack deadenylase activity and may regulate catalytic functions of the complex (6, 33, 34). Individual deletion of CNOT2, CNOT3 and CNOT10 destabilized the complex and caused degradation of other subunits (6, 33, 34). Thus, these CNOT subunits evidently also contribute to the integrity of the whole CCR4-NOT complex. In addition, CNOT2 and CNOT3 form a heterodimer that recruits RNA-binding proteins to the CCR4-NOT complex (35, 36). Moreover, *in vitro* reconstitution experiments showed that the CNOT2-CNOT3 heterodimer maximizes the deadenylase activity and poly (A) selectivity of the CCR4-NOT complex (37).

Ablation of genes encoding individual subunits of the CCR4-NOT complex revealed its roles in various physiological functions. For instance, CNOT3 is required for postnatal liver functions (38), pancreatic β cell function and identity (39), maintaining cardiac homeostasis (40), and bone resorption (41). CNOT7, a catalytic subunit, has non-redundant functions in spermatogenesis (42, 43). In addition, some studies showed a requirement of deadenylation induced by the CCR4-NOT complex in early lymphocyte development (3, 4, 6).

## Functions Of The Ccr4-Not Complex In Lymphocyte Development

Early B cell differentiation has been widely studied (44, 45). Pro-B cells derived from common progenitor cells differentiate into pre-B cells and subsequently immature B cells. In the transition of pro-B cells to pre-B cells, generation of the immunoglobulin (Ig) μ heavy chain assembled from variable (V_H_), diversity (D_H_), and joining (J_H_) gene segments in pro-B cells is essential. Together with surrogate light chains, Igμ heavy chains form a precursor B cell receptor (pre-BCR). Pre-BCR signaling terminates the V(D)J recombination and induces rapid proliferation. After quiescence is re-established by later signaling from pre-BCR, recombination of light chains occurs for further differentiation of pre-B cells into immature B cells.

**Figure 1:**
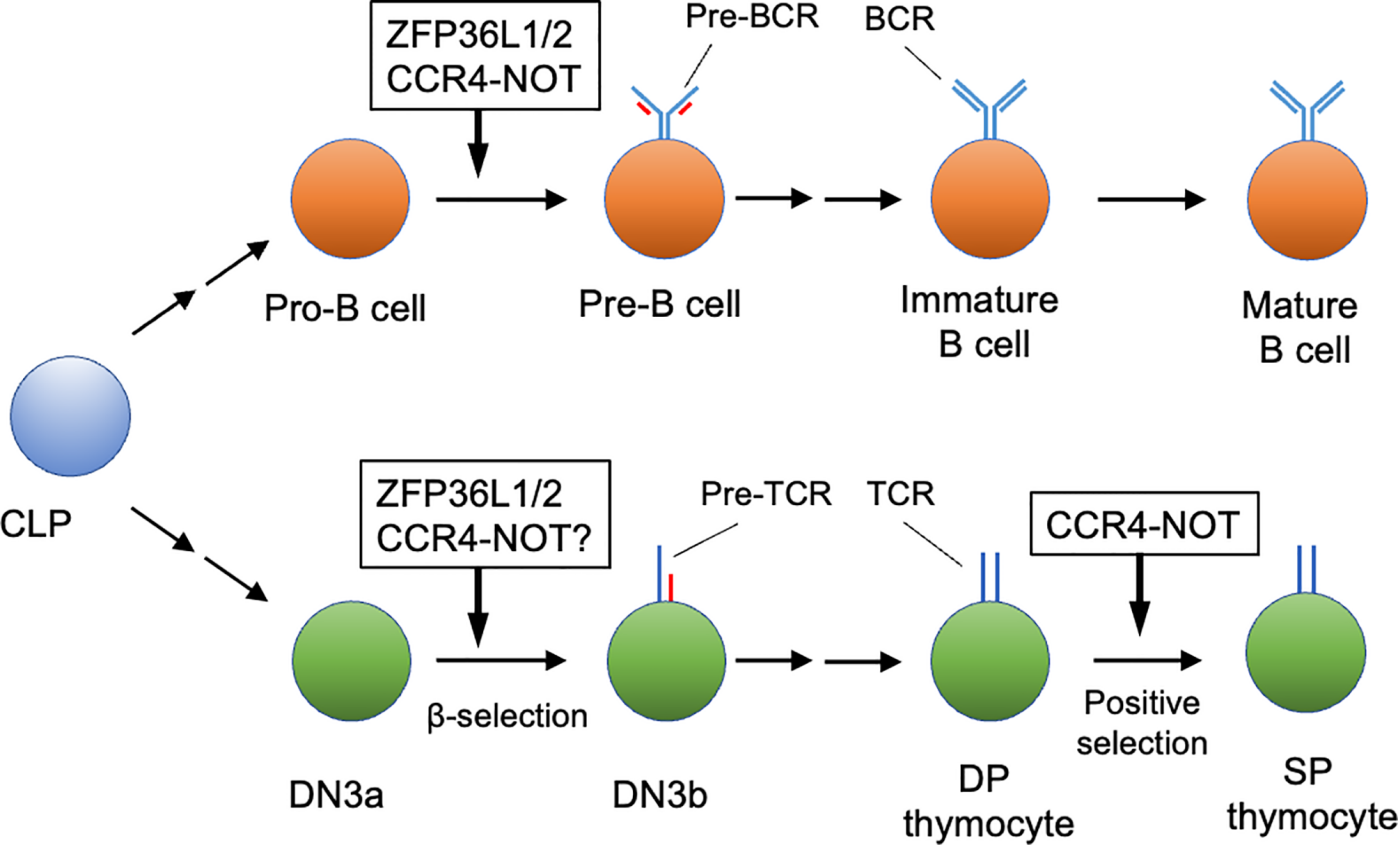
Early lymphocyte development regulated by the CCR4-NOT complex. The CCR4-NOT complex is required for differentiation of pro-B cells to pre-B cells and positive selection of DP thymocytes. ZFP36L1 and ZFP36L2 (ZFP36L1/2) regulate the pro-B cell to pre-B cell transition and β-selection of the DN3 thymocyte stage. Involvement of the CCR4-NOT complex in β selection has not been verified yet. Some differentiation stages were omitted for simplicity. CLP, common lymphoid progenitor; DN, CD4 CD8 double negative thymocyte; DP, CD4 CD8 double positive thymocyte; SP, CD4 or CD8 single positive thymocytes.

In B cell development, CNOT3 protein is up-regulated during differentiation from pro-B cells to pre-B cells (3). Two studies reported a requirement for CNOT3 in B cell development (3, 4). Inoue et al. (3) showed that B cell-specific deletion of *Cnot3* in mice with *Mbl*1-Cre deleter resulted in a severe reduction of pre-B cell differentiation thereafter. Moreover, rearrangements resulting from joining of distal variable gene segments of the Ig heavy chain (*Igh*) gene (V_H_) to its diversity and joining (D_H_ J_H_) gene segments were impaired, although the proximal V_H_ to D_H_ J_H_ rearrangement and D_H_ to J_H_ rearrangement were not affected. Mechanistically, the CCR4-NOT complex mediates deadenylation of *Trp53* mRNA coding p53 protein, reducing its transcript level in pro-B cells. *Cnot3* deletion caused up-regulation of p53, thereby causing an increase in the expression level of pro-apoptotic genes regulated by p53. Interestingly, deletion of the *p53* gene partially rescued the defect of pro-B cell differentiation due to the CNOT3 deficiency, but it did not rescue the failure of the distal V_H_ to D_H_ J_H_ rearrangement. Thus, CCR4-NOT complex-mediated RNA decay ensures gene rearrangement in pro-B cells through p53-independent mechanisms and prevents abnormal apoptosis in both p53-dependent and independent mechanisms during the pro-B cell to pre-B cell transition (**Figure 1**).

Yang et al. also reported a defect in the transition from pro-B cells to pre-B cells due to deletion of the *Cnot3* gene (4). Because *Cnot3*-deletion throughout the body causes embryonic lethality, the tamoxifen-inducible Cre-driver mouse strain in addition to *Mbl1*-Cre mice was used. Even though both mouse lines showed pro-B cell arrest, the effect was more severe in the *Cnot3* deletion in *Mbl1*-Cre. This difference seems to be due to genotoxic stress from excess nuclear accumulation of CRE protein, driven by the *Mbl1*-promoter. Notably, CNOT3 interacts with Early B-cell factor 1 (EBF1), which is critical for establishing B cell lineages. Mutation of a histidine residue in the DNA-binding domain (DBD) of EBF1 abolished its binding to CNOT3. Moreover, transduction of DBD mutant protein rescued the differentiation defect of EBF1-deficient B progenitors less efficiently *in vitro* compared to wild-type protein, suggesting that the interaction between CNOT3 and EBF1 is critical for B cell differentiation. Thus, the CCR4-NOT complex may function in both mRNA decay and transcription in pro-B cell differentiation. However, it is still not clear whether these two events are coupled and whether they influence each other.

Early development of conventional T cells occurs in the thymus (46). Briefly, T cell progenitors from bone marrow differentiate into CD4^–^CD8^–^ (DN) thymocytes. In the DN thymocyte stage, recombination of TCRβ chain genes and their selection occurs. DN thymocytes then differentiate into CD4^+^CD8^+^ (DP) thymocytes. After completing TCRα chain recombination, DP thymocytes undergo positive selection to test the quality and specificity of the TCR αβ complex. Positively selected DP thymocytes differentiate into CD4 or CD8 single-positive (SP) cells. In the process of positive selection, DP and SP thymocytes are further separated by expression levels of surface CD3 and CD69 (47). Before positive selection, DP thymocytes express low levels of CD69 and CD3. During positive selection, CD3 and CD69 expression are up-regulated by TCR signaling. After completion, the expression level of CD3 persists, but surface CD69 expression is down-regulated.

A recent study revealed the activity of the CCR4-NOT complex in positive selection (**Figure 1**). In thymic T cell development, some protein subunits of the CCR4-NOT complex (CNOT1, 2, 3 and 6) are up-regulated in the transition from DN thymocytes to DP thymocytes (6). T-cell-specific deletion of the murine *Cnot3* gene by the CD4-Cre deleter caused a severe developmental defect of CD4 and CD8SP thymocytes in the thymus. Specifically, the *Cnot3* deletion caused a severe reduction of CD69^hi^CD3^hi^ cells and CD69^lo^CD3^hi^ cells, but did not influence pre-selected CD69^lo^CD3^int^ thymocytes, suggesting that the CCR4-NOT complex is required during positive selection. mRNAs encoding pro-apoptotic molecules, DAB2-interacting protein (DAB2IP) (48) and BCL2-binding component 3 (BBC3) (49), were up-regulated and their polyA tails were elongated in *Cnot3*-deficient thymocytes during the course of positive selection. Moreover, transduction of anti-apoptotic Bcl-2 protein into *Cnot3*-deficient bone marrow progenitor cells rescued the developmental defect of thymocytes caused by *Cnot3*-deletion. Thus, by trimming their polyA tails, the CCR4-NOT complex promotes degradation of mRNAs encoding these pro-apoptotic proteins, which are up-regulated during positive selection. This regulation by the CCR4-NOT complex is necessary to prevent abnormal apoptosis during positive selection. Interestingly, up-regulation of *Dab2ip* resulting from *Cnot3* deletion occurred in the CD69^lo^CD3^int^ and CD69^hi^CD3^hi^ stages, but not in the CD69^lo^CD3^hi^ stage. In contrast, *Bbc3* was up-regulated from the CD69^hi^CD3^hi^ stage in the absence of CNOT3. Thus, the CCR4-NOT complex may prevent thymocyte apoptosis during two distinct stages of positive selection *via* two different mechanisms.

## Upstream Events Leading To Ccr4-Not-Mediated Rna Degradation

**Figure 2:**
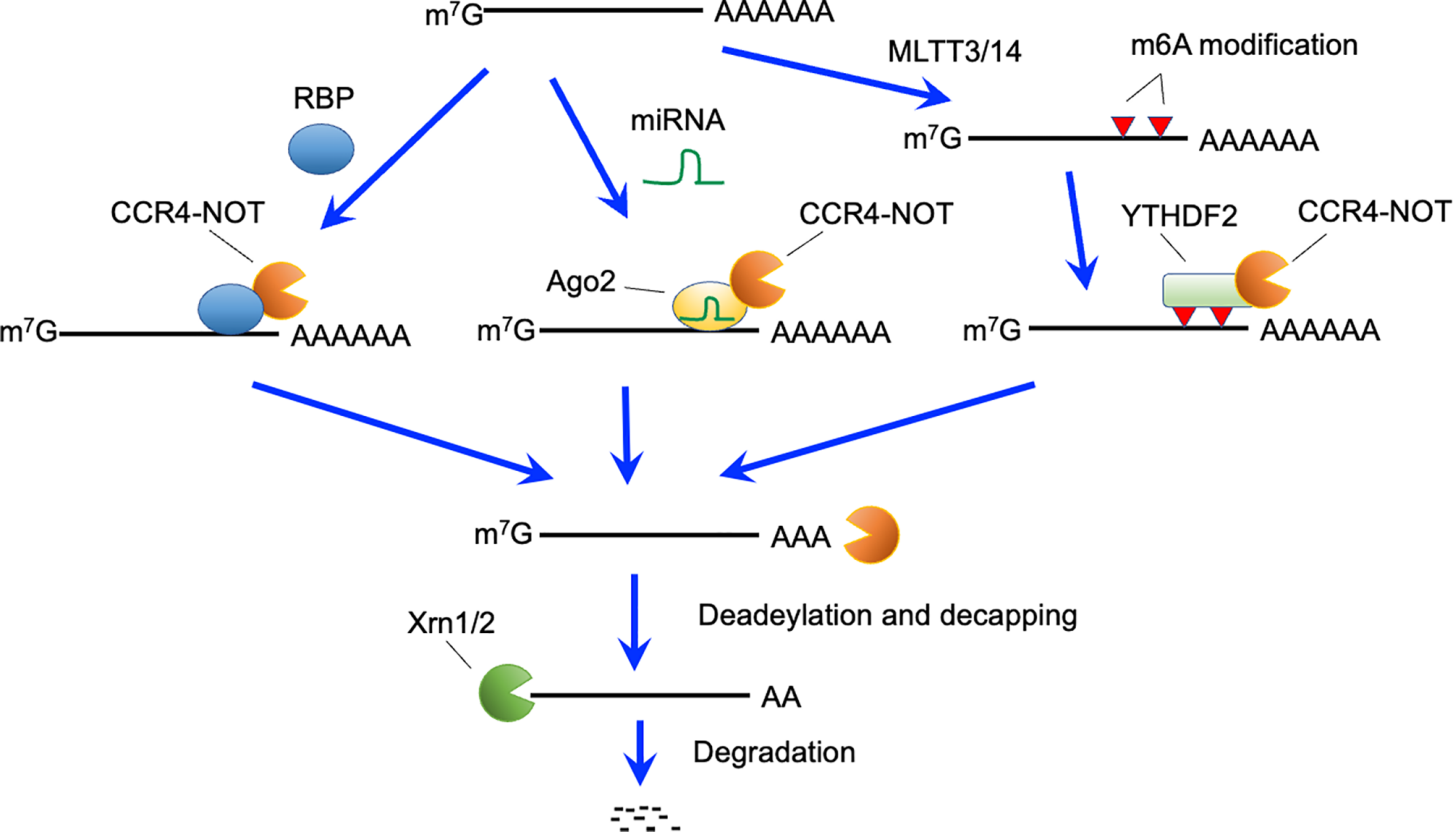
Active RNA decay pathways mediated by the CCR4-NOT complex. The CCR4-NOT complex can be recruited by RNA binding proteins (RBP), miRNA-Ago2 complex, and YTHDF2 bound to N6-methyladeonsine, which is generated by the METTL3 and METTL14 methyltransferase complexes. The CCR4-NOT complex deadenylates polyA tails of recruited mRNAs. After deadenylation, 5’-decapping enzymes are recruited and eliminate the cap structure. Finally, 5’-exonucleases (Xrn1 and 2) causes degradation of target mRNAs.

Several mechanisms reportedly trigger RNA decay by recruiting the CCR4-NOT complex to target mRNAs (**Figure 2**). ZFP36 family proteins, including ZFP36, ZFP36 ring finger protein-like (ZFP36L) 1 and ZFP36L2, bind to specific sequences in 3’ untranslated regions (UTRs) of mRNAs (50, 51). The CCR4-NOT complex is recruited by ZFP36L1 and ZFP36L2 (28), and initiates deadenlyation of mRNAs bound to ZFP36L1 and ZFP36L2, thereby promoting decay of targeted mRNAs. B cell-specific deletion of both *Zfp36l1* and *Zfp36l2* genes caused a severe reduction of cellularity from the pre-B cell stage onward (5). Furthermore, *Igμ* chain expression in pro-B and early pre-B cells was reduced in these mutant mice. Thus, ZFP36L1 or ZFP36L2 is required for differentiation of pro-B cells into pre-B cells and recombination of *Igh* in early B cells (**Figure 1**). Notably, phenotypes of these mutant mice were quite similar to those of B-cell-specific *Cnot3*-deficient mouse lines. Transcriptome analysis of late pre-B cells in *Zfp36l1* and *Zfp36l2* doubly-deficient mice showed an increase in the expression level of several cell-cycle related genes. Consistently, cell cycle analysis showed that pro-B cells in S phase were significantly increased and that those in G0 phase were severely reduced by depletion of ZFP36L1 and ZFP36L2. Progression of V(D)J recombination requires cellular quiescence because expression of RAG2 protein is restricted to G0 and G1 phase (52–55). Thus, ZFP36L1 and ZFP36L2 redundantly suppress cell cycle progression in early B cell stages *via* degradation of mRNAs encoding cell cycle-related genes, which may be required to promote V(D)J recombination. Putative target mRNAs of ZFP36L1 were upregulated in *Cnot3*-deficient pro-B cells (3). In addition, as described, CNOT3 deficiency resulted in failure of V(D)J recombination in pro-B cells in a p53-independent manner (3). Overall, these findings suggest that ZFP36L1 or ZFP36L2 recruits the CCR4-NOT complex and leads to degradation of mRNAs encoding cell cycle-promoting genes, thereby regulating cell cycle entry and exit to promote progression of V(D)J recombination. Because *Zfp36l1* and *Zfp36l2* genes are expressed throughout B cell development, there may be up-stream events activating ZFP36L1 and ZFP36L2, or promoting recruitment to their target mRNAs.

In addition to B cell development, ZFP36L1 and ZFP36L2 regulate early T cell differentiation. As in B cells, *Zfp36l1* and *Zfp36l2* genes are expressed throughout thymocyte development. Mice in which both ZFP36L1 and ZFP36L2 were depleted in early T cell progenitors developed T cell acute lymphoblastic leukemia (56). Importantly, V(D)J recombination of TCRβ gene was defective in DN thymocytes of these doubly-deficient mice. Thus, DN thymocytes in mutant mice by-pass the β-selection checkpoint without expression of TCRβ and are converted into T lymphoblasts. Mechanistically, the ZFP36L1- and ZFP36L2-dependent RNA decay pathway appeared to inhibit up-regulation of genes involved in DNA damage-response and cell proliferation (57). This suppression appeared crucial to induce cellular quiescence required for V(D)J recombination in DN thymocytes. Considering that V(D)J recombination occurs in both DN thymocytes and pro-B cell stages, functions of ZFP36L1 and ZFP36L2 may be similar in these two cell types (**Figure 1**). Although this may imply a requirement for CCR4-NOT complex-dependent RNA decay in the DN stages, involvement of the CCR4-NOT complex in V(D)J recombination of DN thymocytes has not been addressed yet.

Roquin family proteins, ROQUIN-1 and ROQUIN-2, can also recruit the CCR4-NOT complex in the 3’-UTRs of target mRNAs (58). T cell-specific deletion of both *Rc3h1* and *Rc3h2* (encoding ROQUIN-1 and ROQUIN-2, respectively) genes resulted in enhanced helper T cell activation and follicular helper T cell differentiation with spontaneous inflammation (10). Puzzlingly, these phenotypes have not been observed in T-cell-specific *Cnot3*-deficient mice. However, because T-cell-specific deletion of *Cnot3* severely impaired mature T cell development in the thymus, the phenotype that characterized the later T cell stage needs to be clarified. Alternatively, other mRNA decay systems may function downstream of these ROQUIN family proteins.

Among epigenetic modifications of mRNA, the N6-methyl adenosine (m6A) modification in RNA is intensively studied (59, 60). The m6A modification is recognized by the YT521-B homology domain-containing family 2 that recruits the CCR4-NOT complex (61) (**Figure 2**). Thus, m6A modification can lead to CCR4-NOT complex-mediated RNA decay. The N6-methylation of adenosine is catalyzed by methyl transferase (METTL) 3 and METTL14 complexes (62). CD4-Cre-mediated deletion of the *Mettl3* gene suppressed homeostatic proliferation of peripheral CD4-positive T cells under lymphopenic conditions (63). *Socs* mRNA degradation induced by interleukin 7-signaling, which is required for proliferation and differentiation of naïve T cells, was impaired in these mice. Notably, again, the link between m6A modification and CCR4-NOT complex-mediated mRNA decay is still obscure in T cells because of the severe reduction of mature naive T cells in T cell-specific *Cnot3*-deficient mice.

miRNAs form complexes with Argonaute family proteins on their target sequences (64, 65). The miRNA- Argonaute family complex recruits the CCR4-NOT complex or the PAN2-PAN3 complex, leading to degradation of target mRNAs (64, 65). Conditional depletion of Dicer, an enzyme critical for miRNA generation (66) in early B cells inhibited the transition of pro-B to pre-B cells, suggesting a requirement of miRNA for this differentiation stage (67). This phenotype is similar to that in mice deficient for CNOT3. However, the Dicer deficiency did not impair the V(D)J recombination reaction, whereas generation of an antibody repertoire was disturbed (67). Thus, the miRNA-CCR4-NOT complex axis in early B cells still needs to be clarified.

Although deletion of the *Dicer* gene in early thymocytes by the *Lck*-Cre deleter caused a severe reduction in cellularity of thymocytes, the percentage of each thymocyte fraction was not affected (68). Moreover, *Dicer* deletion by *CD4*-Cre showed normal thymocyte number and percentages of thymocyte subsets (69). Overall, miRNA-dependent mRNA decay through recruitment of the CCR4-NOT complex might not be essential for early thymocyte development and selection.

## Concluding Remarks

Rapid, simultaneous and dynamic transcription of several mRNAs occurs during cell differentiation for generation of functional proteins. In addition, protein abundance must be precisely regulated to avoid deleterious cellular consequences. Regulation of mRNA levels should be an efficient way to control protein expression levels because one mRNA molecule generates about 3000 protein molecules on average in mammalian cells (70). However, once proteins are produced, RNA decay mechanisms should be less effective than direct protein degradation for regulating protein levels. Accordingly, it is likely that RNA decay-dependent regulation of protein concentrations by the CCR4-NOT complex is most effective when cells receive signals initiating production of large numbers of mRNA transcripts, such as during lymphocyte development.

It is likely that ZFP36L family proteins recruit the CCR4-NOT complex to suppress cell-cycle-related genes and DNA damage-responsive genes during early lymphocyte differentiation. In contrast, upstream events of CCR4-NOT complex-dependent regulation of thymic positive selection remain unknown. Moreover, possible upstream mechanisms of the CCR4-NOT complex, i.e., ROQUIN family, m6A modification, are necessary to regulate differentiation and functions of lymphocytes in later differentiation stages. Therefore, it is also important to address whether CCR4-NOT complex-induced RNA decay controls these lymphocyte differentiation and functions.

Finally, given that recent studies on human disease progression and onset by dysregulation of RNA decay systems (71–74), understanding RNA decay mechanisms would be beneficial for developing therapies and preventive measures against such diseases.

## Author Contributions

TA wrote the first draft of the manuscript and TY critically reviewed it. All authors contributed to the article and approved the submitted version.

## Conflict of Interest

The authors declare that the research was conducted in the absence of any commercial or financial relationships that could be construed as a potential conflict of interest.
